# Cystoid Macular Edema Induced by Low Doses of Nicotinic Acid

**DOI:** 10.1155/2013/713061

**Published:** 2013-04-11

**Authors:** Daniela Domanico, Carmela Carnevale, Serena Fragiotta, Francesca Verboschi, Simona Altimari, Enzo Maria Vingolo

**Affiliations:** ^1^Department of Ophthalmology, S.M. Goretti Hospital, Via G. Reni, 04100 Latina, Italy; ^2^Department of Ophthalmology, Sapienza University of Rome, Polo Pontino, A. Fiorini Hospital, Via Firenze, 04019 Terracina, Italy; ^3^Department of Sense Organs UOC B, Policlinico Umberto I, Sapienza University of Rome, Viale del policlinico 155, 00161 Rome, Italy; ^4^Department of Ophthalmology, San Giovanni Evangelista Hospital, Tivoli, Italy

## Abstract

Cystoid macular edema (CME) is a condition that involves the macula, causing painless vision loss. In this paper, we report a case of niacin-induced bilateral cystoid macular edema (CME) in a middle-age woman taking low dose of niacin (18 mg of nicotinic acid). Optical coherence tomography (OCT) showed retinal thickening and cystoid spaces in both eyes, whereas fluorescein angiography (FA; HRA 2, Heidelberg Engineering) revealed the absence of fluorescein leakage also in later phases. Four weeks after discontinuation of therapy there were a complete disappearance of macular edema at funduscopic examination and an improvement of visual acuity in both eyes. Furthermore OCT showed a normal retinal profile in both eyes. In our opinion considering the wide availability of niacin, medical monitoring and periodical examination should be considered during niacin administration. To our knowledge, this is the first report in the literature that described the very low-dose niacin-induced bilateral niacin maculopathy.

## 1. Introduction

Cystoid macular edema (CME) is a disorder which involves the central retina, caused by an accumulation of extracellular fluid in the macular region with secondary formation of multiple cystic spaces [1].

The edema affects outer plexiform and inner nuclear layers of the retina and is generally related to permeability changes in blood-retinal barrier that limits the intraretinal movements of plasmatic components and maintains a right homeostasis.

Various conditions can cause cystoid maculopathy such as retinal vascular diseases, intraocular inflammatory diseases, ocular surgery especially cataract extraction, hereditary retinal dystrophies, and topical or systemic assumption of drugs [2, 3]. 

In this paper we report the case of a healthy woman that, after a daily assumption of 18 mg of nicotinic acid, has developed a cystoid macular edema in both eyes.

## 2. Case Presentation

A 53-year-old female (P.P.) was admitted to our Department of Ophthalmology, Sapienza University of Rome, Polo Pontino, for a sudden decrease of visual acuity in both eyes, blurred vision, and metamorphopsia.

She had a medical history of hypertension and type 2 diabetes mellitus well controlled with oral hypoglycemic agents. In the past she had never presented with any vision trouble, previous history of ophthalmological diseases, or ocular surgery. Plasma cholesterol levels slightly higher than the normal range were incidentally found on a routine blood testing. Therefore her general practitioner prescribed her dietary changes, lifestyle modifications, and oral nutritional supplement containing 18 mg of niacin per day (Table 1).

One month before the start of therapy she had undergone a complete ophthalmologic examination: the best-corrected visual acuity (BCVA) was 0.0 logMAR in both eyes; results of slit-lamp examination were unremarkable; no signs of diabetic retinopathy or hypertensive retinopathy were reported on funduscopic examination.

**Figure 1:**
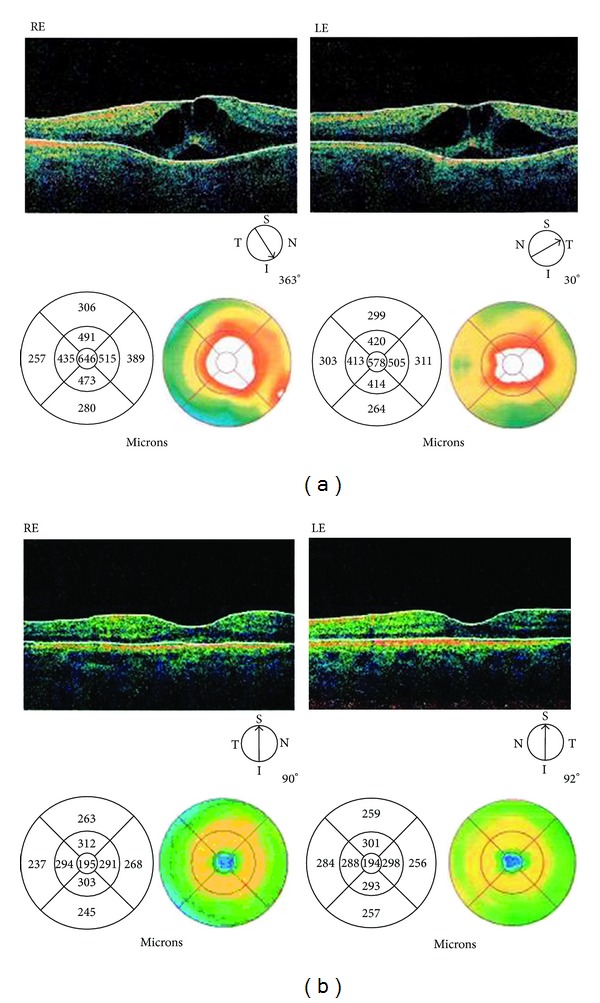
(a) Stratus OCT during niacin treatment. Abnormal retinal profile with the presence of hyporeflective cystoid spaces in the outer plexiform and inner nuclear layers in both eyes. Central macular thickness (CMT) was 646 microns in RE and 578 microns in LE. (b) Stratus OCT four weeks after discontinuation of therapy. Normal retinal thickness with complete disappearance of the hyporeflective cystoid spaces. Central macular thickness (CMT) was 195 microns in RE and 194 microns in LE.

Her visual disturbances appeared 4 weeks after starting nutritional supplement. A complete ophthalmologic examination was performed: BCVA was 0.39 logMAR in the right eye (RE) and 0.52 logMAR in the left eye (LE); the anterior segment examination was normal, pupil reflexes were evoked, crystalline lenses was clear, and in situ intraocular pressure (IOP) measured by Goldman applanation tonometry was 16 mmHg in both eyes. Dilated funduscopic evaluation confirmed the absence of diabetic or hypertensive retinopathy but revealed the presence of bilateral macular edema. In order to confirm and quantify macular edema optical coherence tomography (Stratus OCT, Carl Zeiss Meditec, Dublin, CA, USA) was performed. OCT showed an alteration of retinal profile with increasing of retinal thickness and the presence of hyporeflective cystoid spaces in the outer plexiform and inner nuclear layers in both eyes. Central macular thickness (CMT) was 646 *μ*m in the right eye and 578 *μ*m in the left eye (Figure 1(a)). 

**Figure 2:**
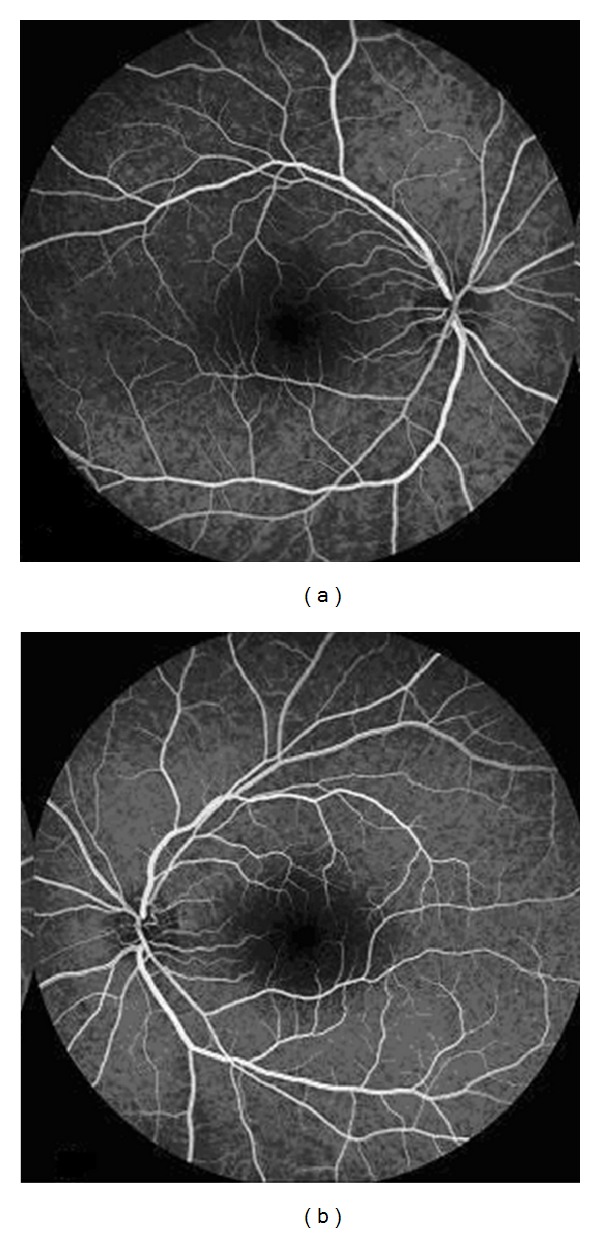
Fluorescein angiography of the right (a) and left (b) eyes nine minutes after injection of fluorescein that revealed the absence of fluorescein leakage typical of cystoid macular edema induced by nicotinic acid in both eyes.

Fluorescein angiography (FA; HRA 2, Heidelberg Engineering) revealed the absence of fluorescein leakage also in later phases (Figures 2(a) and 2(b)).

We advised her to discontinue the assumption of dietary supplement and to make another followup after four weeks. 

Four weeks after discontinuation of therapy, she underwent a complete ophthalmologic examination. She, as ordered, stopped taking the dietary supplement, and she confirmed to have monitored her blood glucose levels and not changed her diet or lifestyle. 

BCVA was 0.04 logMAR in both eyes; at funduscopic examination the macular reflex was evoked with complete disappearance of macular edema; optic nerve head and vessels by size and course were normal in both eyes.

OCT showed a normal retinal profile with the absence of cystoid spaces; central macular thickness (CMT) was reduced to 195 *μ*m in the right eye and 194 *μ*m in the left eye (Figure 1(b)).

## 3. Discussion

Niacin (nicotinic acid, vitamin B3, vitamin PP), one component of the dietary supplement taken by the patient, is a vitamin preparation usually used for the treatment of lipid disorders. The mechanism of action affects the plasma levels of the apolipoproteins (apo) A and B. The decrease of Apo B-100 causes a reduction of very-low-density lipoprotein (VLDL), low-density lipoprotein (LDL), and lipoprotein(a) [Lp(a)], whereas the decrease of hepatic clearance of Apo A-I raises the level of plasma high-density lipoprotein (HDL) [4]. Nicotinic acid inhibits lipolysis reducing plasma fatty acids necessary to triglyceride (TG) synthesis. Furthermore, niacin therapy reduces the risk of cardiovascular diseases and the progression of coronary atherosclerosis by decreasing circulating levels of fibrinogen and increasing of plasminogen-activating factor (PAF) [5].

Pharmacological doses of niacin can cause different adverse effects. The most frequent is the prostaglandin-mediated vasodilatation of the small blood vessels resulting in cutaneous flushing, warmth, and pruritus, usually involving the face, neck, and upper trunk. The duration of the reaction is variable from a few minutes to 2–4 hours, and with continuation of therapy the symptoms become less intense until to disappear. Dry skin and cutaneous hyperpigmentation (acanthosis nigricans) are also commonly reported [4, 6]. Clinical studies suggest that niacin can raise the plasma levels of uric acid and reduce the glucose tolerance [7].

Fraunfelder et al. reported that 3 g or more per day of nicotinic acid could cause many ocular side effects such as blurred vision, eyelid edema, toxic amblyopia, proptosis, loss of eyelashes or eyebrow, superficial punctate keratitis, and cystoid macular edema, which represents the most serious ocular complication. All these adverse effects are reversible with discontinuation of niacin therapy [8, 9].

In 1973, Donald M. Gass reported the case of three patients (mean age 50 years) with hypercholesterolemia that, after high doses of niacin (3–6 g/day), developed a sudden decrease of central vision due to cystoid macular edema in both eyes [10].

Nicotinic acid maculopathy affects more frequently men, with a male : female ratio of 10 : 1 [11]. A retrospective study showed that only 2 of 300 patients developed CME [12]. Furthermore, although niacin maculopathy generally occurs at doses of 3 g per day or more, it has been seen in patients taking as little as 1.5 g per day [9]. 

In our case a very low dose of niacin was administered (18 mg of nicotinic acid) and niacin maculopathy appears 4 weeks later with a complete resolution after discontinuation of drug, confirming the relationship between drug administration and macular edema appearance. Funduscopic examination showed bilateral macular edema, but no signs of diabetic or hypertensive retinopathy excluding the presence of other factors that may contribute to the capillary dysfunction associated with edema onset. 

At funduscopic examination the foveomacular area has a peculiar aspect: the foveola takes on a bright yellow hue, similar to an exudate. Cysts primarily involve the foveal area and they are smaller than those seen in postsurgical or inflammatory cystoid macular edema [12]. 

FA and OCT are useful to confirm the diagnosis. FA showed the absence of fluorescein leakage or vascular alteration, whereas OCT confirms the presence of cystic hyporeflective spaces in macular region, as reported in the literature. Indeed previous studies reported that the fluorangiographic pattern is atypical. The petaloid appearance caused by fluorescein dye leakage typical of CME is absent. Instead OCT shows the presence of intraretinal cystoid spaces, that appear more numerous and larger in the outer plexiform layer that is in the inner nuclear one [13–15].

 Whether nicotinic acid induced cystoid macular edema is still unknown. A first hypothesis suggests that, in patients with vascular or inflammatory diseases, niacin induces the release of prostaglandins, causing the blood-retinal barrier compromise with extracellular accumulation of fluid in many cystic spaces. This theory is not fully accepted for the absence of fluorescein leakage [16]. A second and the most reliable hypothesis supports that niacin has direct toxic effects on Müller cells, without disruption of the blood-retinal-barrier. Alterations in cellular metabolism cause intracellular fluid increase and swelling of these cells, with secondary formation of cysts between the glial spaces. After the discontinuation of niacin therapy there is a complete regeneration of the Müller cells and their normal function [17, 18]. Moreover Müller cells seem to be affected early in the course of diabetes [19, 20]. In our opinion, in diabetic patients the dysfunction of Müller cells may predispose to the development of macular edema, even at low doses of the drug. Indeed, our patient had a well-controlled type 2 diabetes mellitus, with no signs of diabetic retinopathy. 

To our knowledge this is the first report in the literature of CME caused by very low dose of niacin (18 mg). In our opinion niacin should be used with caution and under medical monitoring or periodic examination. In particular, patients with ocular symptoms, such as blurred vision, decrease of visual acuity, and metamorphopsia, should immediately go to an ophthalmologist and stop the assumption of nicotinic acid. Further studies would be desirable to investigate the safety dose, pathophysiologic mechanisms or predisposing risk factors, and the possible interaction with other agents that may cause niacin maculopathy. 

## Figures and Tables

**Table 1:** Dietary supplement composition.

Nutritional supplement component for tablet	
Niacin	18 mg
Vitamin E	10 mg
Chrome	50 mcg
Various herbal components	560 mg
